# Hepatitis C Infection Patterns at a Tertiary Care Center in New York: A Cross-Sectional Study

**DOI:** 10.7759/cureus.2225

**Published:** 2018-02-26

**Authors:** Ahmed Mahgoub, Talal El Imad, Hassan Al Moussawi, Danial Daneshvar, Fady G. Haddad, Joseph Saabiye, George Abdelsayed, Sherif Andrawes, Liliane Deeb

**Affiliations:** 1 Department of Internal Medicine, Staten Island University Hospital, New York; 2 Department of Medicine, Staten Island University Hospital, New York; 3 Internal Medicine, Northwell Health-Staten Island University Hospital, New York; 4 Department of Gastroenterology and Hepatology, Staten Island University Hospital, New York; 5 Internal Medicine, Staten Island University Hospital, New York; 6 Gastroenterology, New York University; 7 Department of Gastroenterology, Staten Island University Hospital, New York

**Keywords:** hcv, liver disease

## Abstract

Introduction

In the United States, 2.7 to 3.9 million patients are infected with the hepatitis C virus (HCV) with 3,500 new cases reported yearly. According to the Centers for Disease Control and Prevention, HCV was the underlying or contributing cause of death of 19,659 patients in 2014. These facts underscore the need for a better understanding of the scope of this disease. Our epidemiologic study aimed at analyzing the pattern of occurrence of HCV infection at Staten Island University Hospital (SIUH) by evaluating the characteristics of newly infected patients with hepatitis C in 2014. The identified features served to better distinguish the targets for preventive health care in our particular population.

Methodology

A cross-sectional study of all newly diagnosed patients with HCV infections in the year 2014 presenting to SIUH was conducted using International Classification of Disease-9 codes (ICD-9) for hepatitis C. We included all patients with a positive HCV antibody confirmed by polymerase chain reaction testing. Patients were divided into groups according to age to simulate the age groups in the 2013 - 2014 Hepatitis B and C Annual Report of the New York City (NYC) Department of Health and Mental Hygiene published in 2016 (abbreviated to 2014 NYCDOH Report, hereafter). Gender and HCV genotypes were also collected. We compared disease frequency between age groups, gender, and genotype with the results of the 2014 NYCDOH Report.

Results

A total of 378 newly diagnosed HCV cases were identified; 60.05% were men, and 39.95% were women. The rate of infection with genotype 1a was the highest (36. 5%) followed by 1b (25.9%). In women, genotype 1b was predominant (13.76%) versus genotype 1a as the most common in men. The mean age was 54 years for men and 57 years for women. Most cases fell into the 60 to 69-year age group (32.28%), followed by the 50 to 59-year age group (31.48%). More so, all patients 80 years and older were exclusively women.

Conclusions

We found most new HCV infections at SIUH were diagnosed in patients aged 60 to 69 years, and the 2014 NYC DOH Report indicates most new HCV infections occur in patients aged 40 to 59 years. Also, all HCV infections detected in patients older than 80 years of age were found in women. These findings provide a better understanding of the patient demographics for appropriate HCV screening policies. Increased awareness and strict adherence to screening policies in baby boomers and high-risk populations are paramount in order to diagnose HCV infection early, offer therapy, and prevent HCV-related mortality and morbidity.

## Introduction

Hepatitis C is a prevalent disease affecting around 130 to 150 million patients worldwide [1]. In the United States (US), estimates of patients having chronic hepatitis C range between 2.7 and 3.9 million [2]. Hepatitis C is associated with significant morbidity and mortality and is the most common indication for liver transplantation in the US. It has a wide range of clinical presentations, including chronic hepatitis, liver cirrhosis, and liver failure, in addition to a higher risk for hepatocellular carcinoma. Patients with hepatitis C have a 37% increased risk of death, mainly attributed to complications of decompensated liver cirrhosis or progression to hepatocellular carcinoma [3].

Multiple factors affect the rate of deterioration of liver disease in patients with hepatitis C. These factors include the age at which the infection was acquired, gender, hepatitis C virus (HCV) genotype, simultaneous infection with hepatitis B and human immunodeficiency virus (HIV) viruses, alcohol abuse, and exposure to hepatotoxic drugs. Identifying the genotype of hepatitis C is crucial in this perspective because, in addition to its ability to forecast the rate of progression of liver disease, it also predicts the response to the new oral antiviral medications and affects the duration of therapy. There are six genotypes of hepatitis C. HCV genotype 1 is the most prevalent in the US, followed by genotypes 2 and 3. Genotype 3 is known to be more resistant to treatment, and patients infected with this genotype are more prone to complications from liver disease. The epidemiology of hepatitis C varies across the US, and the genotype predominance differs between the states and differs according to the demographic characteristics of the affected population. In view of the heavy burden HCV infection confers on the health care system, it is important to know the directions this widespread disease is taking both locally and at the national level. In this study, we looked at the newly diagnosed HCV cases at Staten Island University Hospital (SIUH) from 2013 to 2014, investigated the demographics, and determined the prevalence of different hepatitis C genotypes. Then, we compared our findings to those reported in the 2013 - 2014 Hepatitis B and C Annual Report of the New York City (NYC) Department of Health and Mental Hygiene published in 2016 (abbreviated to 2014 NYCDOH Report, hereafter) aiming to find a trend or pattern that allows for more and better understanding of the disease dynamics and behavior at the local level in the Staten Island borough [4].

## Materials and methods

We conducted a retrospective longitudinal review of the medical records of patients diagnosed with HCV from 2013 to 2014 at SIUH. Subjects were identified using International Classification of Disease-9 codes for chronic hepatitis C. Only those with positive HCV antibodies confirmed with polymerase chain reaction testing during the study period were enrolled. This study consisted solely of data collection by chart review. A total of 378 patient medical records were reviewed. No direct contact between investigators and patients took place. Thus, informed consent was not required. Investigators collected demographic, medical, and laboratory data from McKesson system electronic health records. The study divided the patients into different age groups for patients aged 0 to 19 years, 20 to 29 years, and so on. The age groups simulate the age groups in the 2014 NYCDOH Report [4]. The gender and the genotypes were also collected. This study looks at the correlation between disease frequency based on gender, genotype frequency, disease frequency based on age groups, genotype frequency in each age group, genotype frequency among male patients versus female patients, genotype frequency in different age groups in the female sex, genotype frequency in different age groups in the male sex, and the mean age of male and female patients with the disease. All gathered data were grouped in one confidential electronic database using REDCap electronic data capture tools (Vanderbilt University, Nashville, TN) and handed to the primary investigator for grouping and primary statistical analysis. The primary outcome of this study was to compare the disease frequency based on gender, genotype, and age groups at SIUH with those reported in the 2014 NYCDOH Report published in 2016 [4].The secondary outcome was to compare the genotype frequency in each age group at SIUH to those reported in the same 2014 NYCDOH Report.

Patient demographics and clinical characteristics were summarized and organized by study group. Descriptive statistics (i.e., number of patients, mean, standard deviation, median, and range) were provided for continuous variables. Frequency distribution and percentages were provided for all categorical variables. Data analysis was performed using the IBM SPSS Statistics for Windows, Version 24.0, released 2016, (IBM Corp. Armonk, NY), with p < 0.05 deemed statistically significant.

## Results

A total of 378 newly diagnosed HCV cases were identified in SIUH in 2014. Male patients comprised 227 (60.05%) cases, and female patients comprised 151 (39.95%) cases.

**Figure 1:**
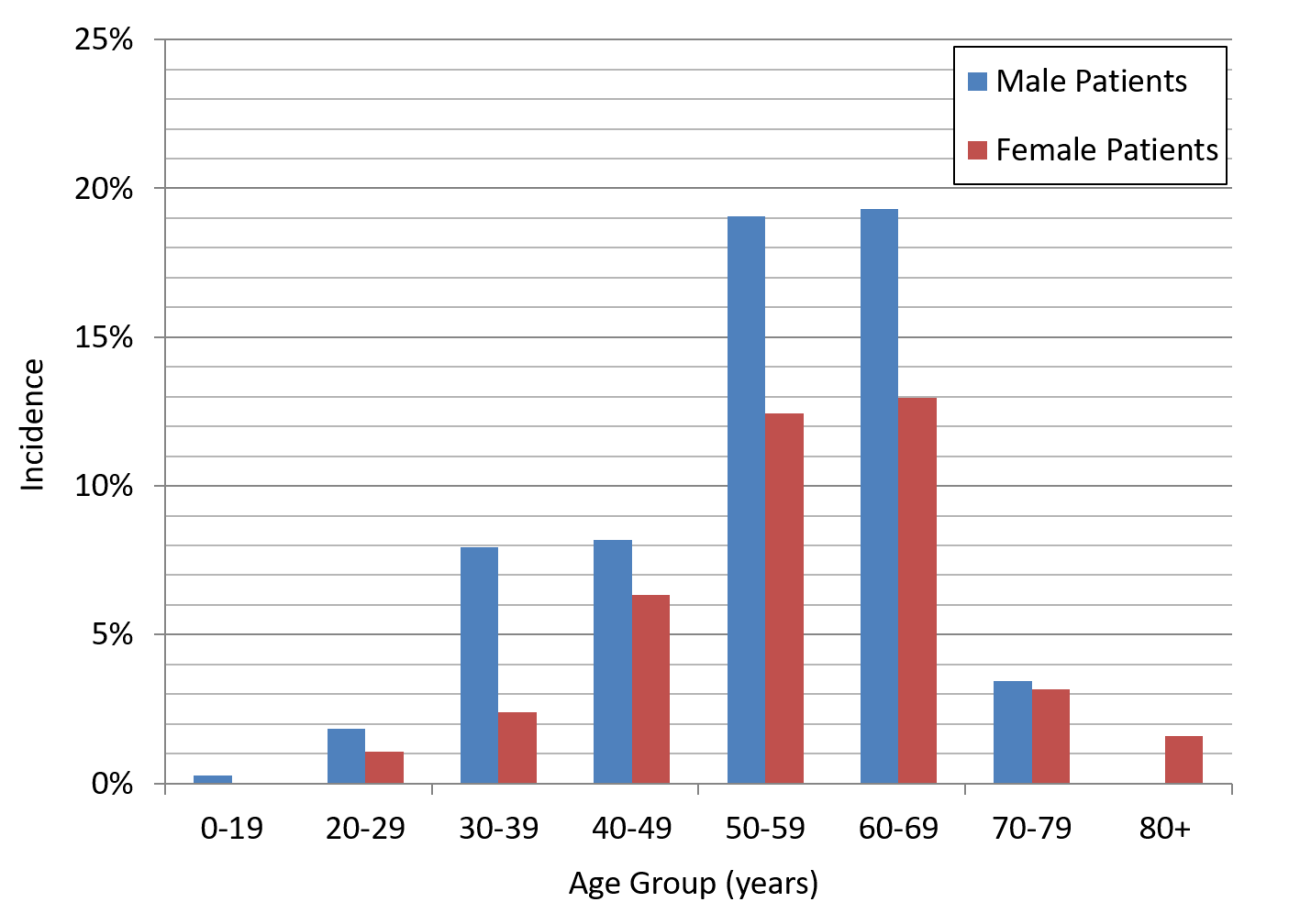
Incidence of HCV cases by sex and age group HCV: hepatitis C virus

In the 2014 NYCDOH Report, male patients represented 60.3% of the cases while 39.6% were female patients. The mean age for male patients was 54 years and 57 years for female patients. The distribution of cases by age groups was as follows: 60 to 69 (32.28%), 50 to 59 (31.48%), 40 to 49 (14.5%), 30 to 39 (10.32%), 70 to 79 (6.61%), 20 to 29 (2.91%), and above 80 (1.59%). The 60 to 69 year age group comprised the majority of cases, both male (32.16%) and female (32.45%) patients, followed by the 50 to 59 year age group (Figure 1).

**Figure 2:**
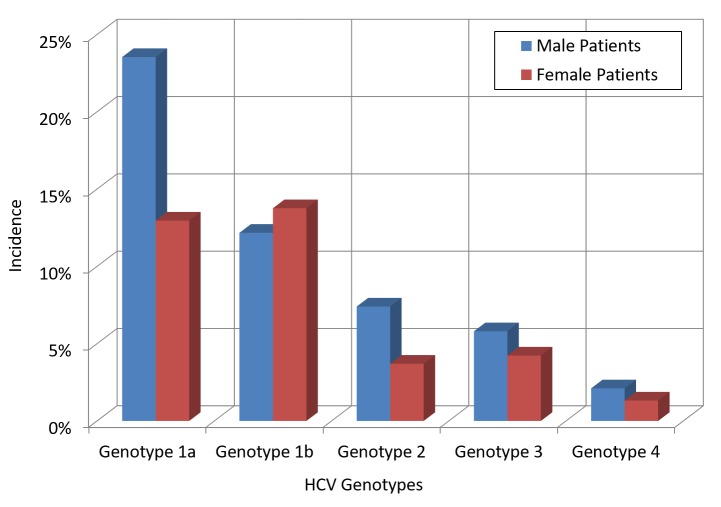
Trend of HCV genotypes by gender HCV: hepatitis C virus

This finding contrasts with the 2014 NYCDOH Report, which showed that most newly diagnosed HCV cases were patients in the 50 to 59 year age group (27%), followed by 22.8% in the 60 to 69 year age group. Regarding the genotypes encountered, genotype 1a was the most prevalent (36.5%), followed by 1b (25.9%), 3a (8.7%), and 2b (7.14%). The most common genotype in female patients was 1b (13.76%), followed by 1a (12.96%) and 3a (3.44%), while the most frequent genotype in male patients was 1a (23.54%), followed by 1b (12.17%) and 3a (5.29%). Of note, genotype 1b was more common amongst female patients (13.76%) than male patients (12.17%) (Figure 2).

**Figure 3:**
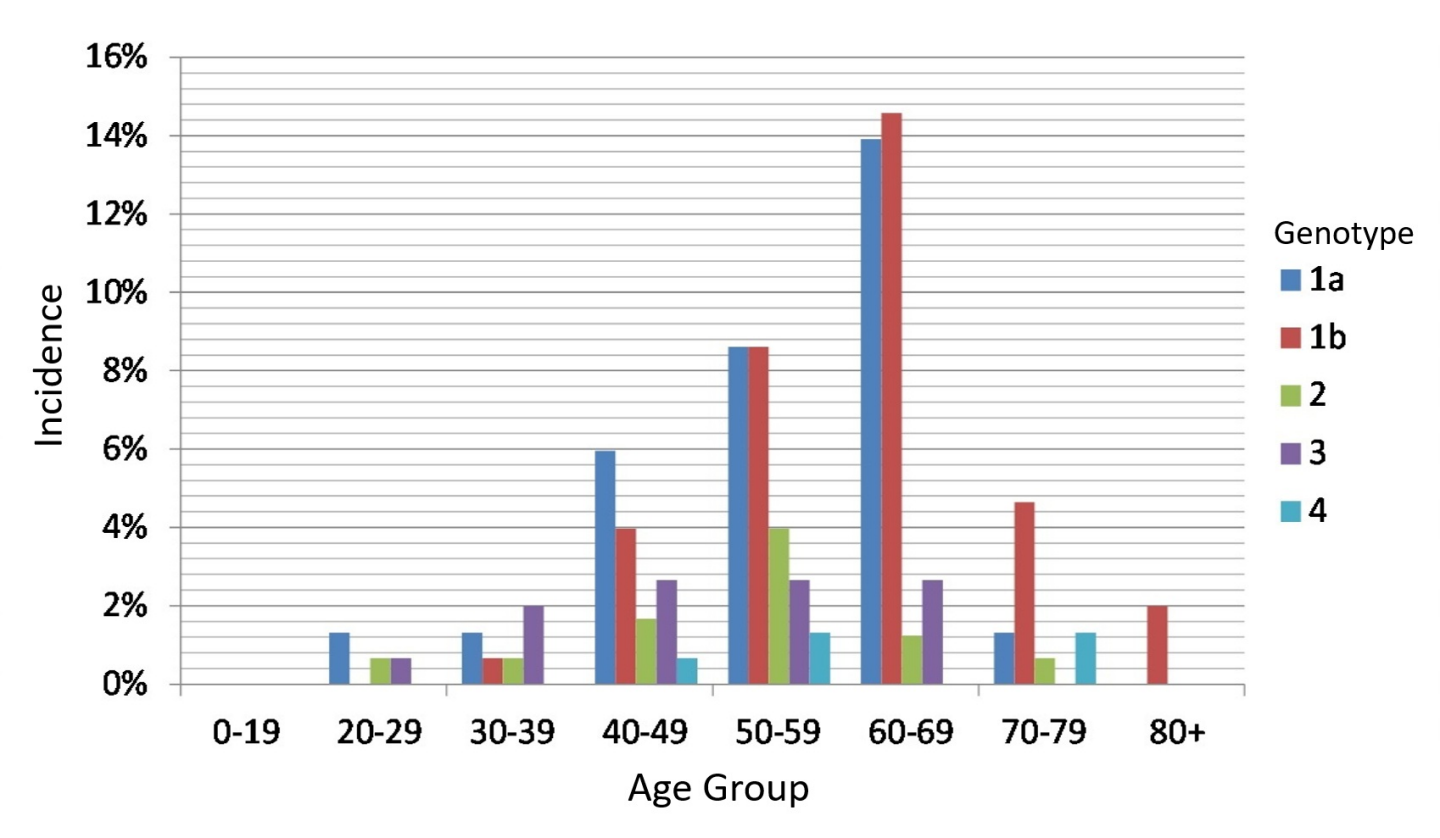
Incidence of HCV genotypes by age group HCV: hepatitis C virus

In female patients, the 30 to 39 year age group had the lowest rate of infection when compared to the same age group in the male patients (2.38% in female patients vs. 7.94% in male patients). Moreover, the most common genotype in female patients for that age group was 3a (30% of the female cases). Notably, there were no reported genotype 3 cases after the age of 69 years in both male and female patients. The highest rise in the number of cases according to age was from the 40 to 49 year age group to the 50 to 59 year age group; it increased by an approximate rate of 116%. In male patients, genotype 1a was always the most common in all age groups above 20 years. In female patients, genotype 1a was the most common in the 40 to 49 year age group, and genotypes 1a and 1b were almost equal in the 50 to 59 year age group. Above the age of 60, genotype 1b became the leading genotype (Figure 3).

When comparing the 60 to 69 year age group to the 70 to 79 year age group, the rate of genotype 1a significantly dropped by nearly 600%. The rate of genotype 1b increased consistently with age in both male and female patients. The number of cases in male and female patients in the 70 to 79 year age group was almost the same. However, after age 79, all cases became exclusively female with genotype 1b.

## Discussion

According to the World Health Organization, HCV represents a global health concern with a prevalence rate of about 71 million patients worldwide; of them, 399,000 die each year. Moreover, the chronicity of this disease and high transmission rates pose additional challenges. Early detection and prompt treatment remain crucial to prevent the development of liver cirrhosis and/or hepatocellular carcinoma [5].

Being the most common blood-borne infection in the US, the Centers for Disease Control (CDC) estimated that 2.7 to 3.9 million Americans have chronic hepatitis C, representing 1.3% of the US population and resulting in about 20,000 deaths per year [4]. Although the HCV mortality rate exceeds HIV mortality [6], the resources devoted to HIV surveillance and treatment surpass those dedicated to HCV. Other reports suggested a prevalence rate of 5.2, higher than what was published by the CDC given that CDC relied on the National Health and Nutrition Examination Survey (NHANES) in estimating the prevalence rate [7]. However, the NHANES survey excluded a considerable number of patients with a high risk of HCV contraction, such as incarcerated individuals, nursing home residents, homeless, hospitalized patients, and individuals on active military duty [8].

In a 2012 study, it was projected that 75% of the HCV patient population were born from 1945 to 1965 [9], and hence, the mandated screening measures targeting that population has become a standard measure for health care maintenance since 2014.

In NYC, it was estimated that the prevalence of chronic HCV infection was about 1.8% of the total population aged more than 19 years and excluded the high-risk population, such as prisoners and intravenous drug abusers [10]. On an annual basis, the NYC Department of Health and Mental Hygiene publishes a hepatitis C report. The report contains detailed epidemiologic data about HCV in NYC, which guides the preventive strategies undertaken by the department.

In our study, we aimed to compare our epidemiologic findings of all reported HCV cases in 2014 at SIUH (the largest health care center in Staten Island) with those published in the 2014 NYCDOH Report.

The 2014 NYCDOH Report showed that Staten Island had the lowest rate (4.1%) of the total reported HCV infections in 2014 [4]. The NYCDOH reported 312 cases in Staten Island, close to our reported number of 378 [4]. Also, the disease frequency based on sex was very similar: female patient frequency was 39.6%, and male patient frequency was 60.3% in the 2014 NYCDOH Report, whereas we found 39.95% of patients were female and 60.05% were male in our study.

In terms of the age groups, both reports showed that the 0 to 19 year age group had the lowest infection rate. However, the 2014 NYCDOH Report showed most cases fell in the 50 to 59 year age group (27%), followed by the 60 to 69 year age group (22.8%). This finding underscores the benefit of the mandated screening for baby boomers (i.e., those aged 50 to 69 years), as shown here that almost 50% of the cases fall in this age group. In our report, we found that the majority of Staten Island cases occurred in the 60 to 69 year age group (32.2%), followed by those in the 50 to 59 year age group (31.4%). With more than 60% of the Staten Island cases diagnosed in patients aged between 50 to 69 years, most Staten Island cases were in the 60 to 69 year age group, which might signal a need to increase our screening measures to catch the bulk of those cases early and thus avoid disease complications.

Regarding the genotype distribution, the 2014 NYCDOH Report noted that genotype 1a was the most common (44.1%), followed by 1b (23.6%), then genotypes 2 and 3. We similarly reported a 36.5% incidence for genotype 1a, followed by 25.9% for 1b; we also found that genotype 1a is the most common in male patients (23.5%), followed by genotype 1b (12.1%). Genotype 1b is the most common in female patients (13.7%), followed by genotype 1a (12.9%). The 2014 NYCDOH Report did not report genotypes per gender.

Our study noted some interesting findings. First, genotype 1b is the most common in patients above the age of 59, and it is the only genotype that exists in patients above the age of 79. This finding underlines the benign course of genotype 1b and its responsiveness to treatment. On the other hand, genotype 3 vanishes from the genotypes map for patients above the age of 69, which might indicate the progressive course of that genotype and its refractoriness to treatment.

Furthermore, our report showed that no male patients survived after the age of 79, and only female patients represented all the cases above that age, most with genotype 1b. This finding may highlight a mortality difference between men and women with HCV, which was indicated in the 2014 NYCDOH Report with 36.2% mortality in women and 63.8% mortality in men. This key finding supports what Inoue et al. and Bakr et al. suggested in that female patients exhibit a higher HCV clearance rate than male patients [11-12]. More studies are warranted to assess this observed effect and examine its etiological nature.

## Conclusions

To a large extent, our epidemiologic data appear to be very consistent with the data reported in the 2014 NYCDOH Report. However, our findings suggest that Staten Island health care facilities should prepare to increase and improve screening strategies in baby boomer patients to shift the main age of diagnosis to the younger age group, thus avoiding a heavy toll of morbidity and mortality. While we do not currently know why only women with HCV survived after the age of 79, our findings add to the very limited data suggesting that women clear the virus more than men.
